# The surgical techniques of transperineal anastomotic urethroplasty for complex posterior urethral stenosis in boys and the long-term follow up outcomes

**DOI:** 10.3389/fped.2023.1009259

**Published:** 2023-03-13

**Authors:** Weidong Zhu, Lujie Song, Yinglong Sa, Yuemin Xu, Qiang Fu

**Affiliations:** ^1^ Department of Urology, Shanghai Sixth People's Hospital Affiliated to Shanghai Jiao Tong University School of Medicine, Shanghai Eastern Urological Reconstruction and Repair Institute, Shanghai

**Keywords:** posterior urethral stricture, pediatric urology, end-to-end urethral anastomosis, traumatic urethral injury, urinary incontinence, erectile dysfunction

## Abstract

**Objective:**

To explored the curative effects of various surgical methods used to treat complicated posterior urethral strictures in boys and the long-term complication.

**Methods:**

We retrospectively studied 28 boys under 14 years of age with complicated posterior urethral strictures treated at our hospital from January 2015 to December 2020. Urethral angiography revealed posterior urethral strictures. Twelve had previously failed urethral surgery; four had urethral fistulae. All underwent end-to-end urethral anastomoses *via* a transperineal, inferior pubic approach. We freed the distal end of the urethra, split the penile cavernous septum, partially resected the lower edge of the pubic symphysis, and rerouted the urethra under a corpus cavernosum to reduce the tension of the urethral anastomosis.

**Results:**

All boys were 2–14 years of age at the time of surgery (mean 6.3 years). The urethral strictures were 3–5.5 cm in length (mean 4.2 cm). Catheters were removed 4 weeks postoperatively. The postoperative follow-up time was 4–72 months (mean 36.8 months). Twenty-four patients exhibited unobstructed urination after a single operation. The maximum urinary flow rate was 15–22 ml/s (average 17.8 ml/s); the success rate was 85.7%. Two patients required second urethral end-to-end anastomoses; urination became normal postoperatively. Two continued to exhibit cystostomies, and two evidenced mild incontinence. Of the six children who have attained puberty, two report erectile dysfunction.

**Conclusion:**

End-to-end urethral anastomosis *via* a transperineal inferior pubic approach is an ideal treatment for posterior urethral strictures in boys. The complications include incontinence and erectile dysfunction, and require long-term follow-up.

## Introduction

Traumatic posterior urethral stenosis in children is a rare condition, but presents a major surgical challenge to pediatric urologist (1). Firstly, children have immature pelvic bones and unstable fractures associated with severely displaced prostatic urethras. Secondly, due to relative intra-abdominal position of a child's bladder there is high incidence of simultaneous bladder neck and sphincter complex injury along with urethral trauma. Lastly, children have smaller pelvic confines, smaller urethral calibre, and greater tissue fragility (2, 3). The previous reports were all summary and discussion of one kind of surgical method for posterior urethral stenosis in children, and there was no long-term follow-up of surgical complications. In this study, various surgical methods of complex posterior urethral atresia in boys and the key points of operation were systematically summarized, It provided more detailed reference for urologists to deal with the complex posterior urethral stenosis in children. Moreover, Preliminary long-term follow-up was also conducted.

In the present study, we present our experience of the surgical technics for the traumatic posterior urethral stenosis in the boys and the preliminary long-term follow-up for postoperative complications.

## Patients and methods

### Patients and preoperative preparation

From January 2015 to December 2020, a total of 28 children younger than 14 years were treated for complicated urethral strictures at our hospital. All were boys with trauma-induced posterior urethral injuries. The mean age was 6.3 years. All underwent preoperative retrograde and voiding cystourethrography and some underwent flexible cystoscopy, revealing posterior urethral strictures or atresia. All underwent end-to-end urethral anastomosis *via* the inferior perineal pubic symphysis after routine preoperative preparation. All underwent urinalysis, urine culture, and sensitivity testing after admission; penicillin or other antibiotics were preoperatively initiated to prevent infection. Povidone-iodine saline irrigation of the bladder and urethra was performed twice daily. The four boys with urethrorectal fistulae were given rectal soapsuds enemas daily for 3 days before surgery.

### Surgical techniques

All patients were placed in the standard lithotomy position after the induction of general anesthesia. Urethroplasty was performed as described previously (4). Briefly, an inverted Y-shaped incision was created in the perineum, the distal bulbar urethra was circumferentially mobilized, and the fibrous tissue between the two ends of the disrupted posterior urethra completely excised until the normal urethral mucosa was fully exposed. For the four patients with urethrorectal fistulae, the entire proximal urethrae were carefully dissected and separated from the rectum after complete dissection of obstructive fibrous scar tissue. The fibrous fistulous tract was completely excised and the fistular margins in the rectum freshened. The rectum was repaired in two layers using 3-0 or 4-0 polygalactin continuous sutures; a pedicled, scrotal sarcoid flap was used for packing. The urethroplasty technique depended on the length of the urethral defect and the extent of dissociation of the distal urethra. We performed a penile septum incision, partial resection of the lower edge of the pubic symphysis, and urethral rerouting under one corpus cavernosum to shorten the distance between the proximal and distal ends of the urethra and achieve a tension-free anastomosis. A 5-0 absorbable thread was used to intermittently suture the distal and proximal urethra (eight stitches). An F8-12 silicone balloon catheter was inserted into the urethra. Dressings were regularly changed and routine anti-inflammatory and hemostatic treatments given. The drainage and urinary catheters were removed 3 days and 4 weeks postoperatively, respectively. The urine flow rate was tested to determine the surgical outcome and the follow-up period required. We telephoned all boys to obtain up-to-date information on long-term complications, including incontinence and erectile dysfunction (ED). No objective ED diagnostic test is available; self-reported ED status may be inaccurate.

## Results

**Figure 1:**
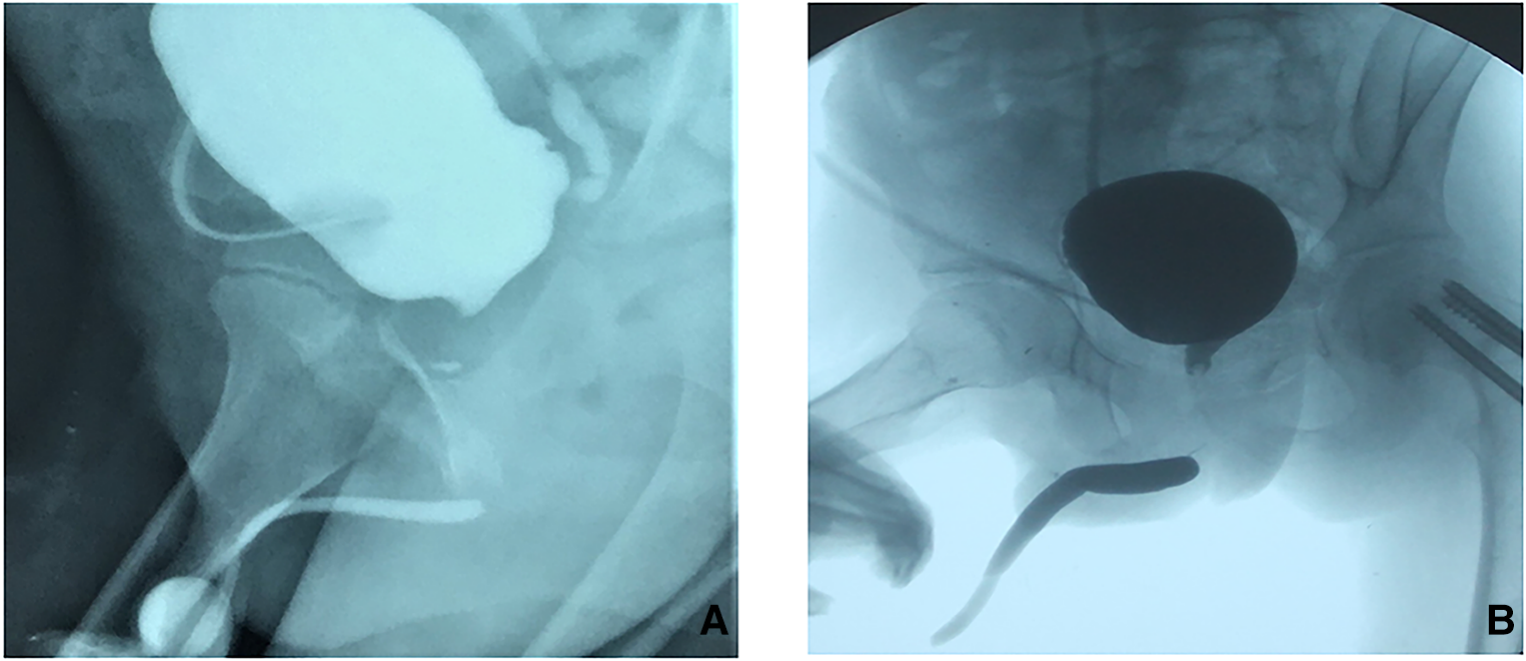
Preoperative cystourethrography of boy with complicated long posterior urethral stricture (**A**): Urethral angiography indicates long posterior urethral stenosis; (**B**): Urethral angiography indicates that the bladder floats up and the proximal opening of the posterior urethra is high.

**Figure 2:**
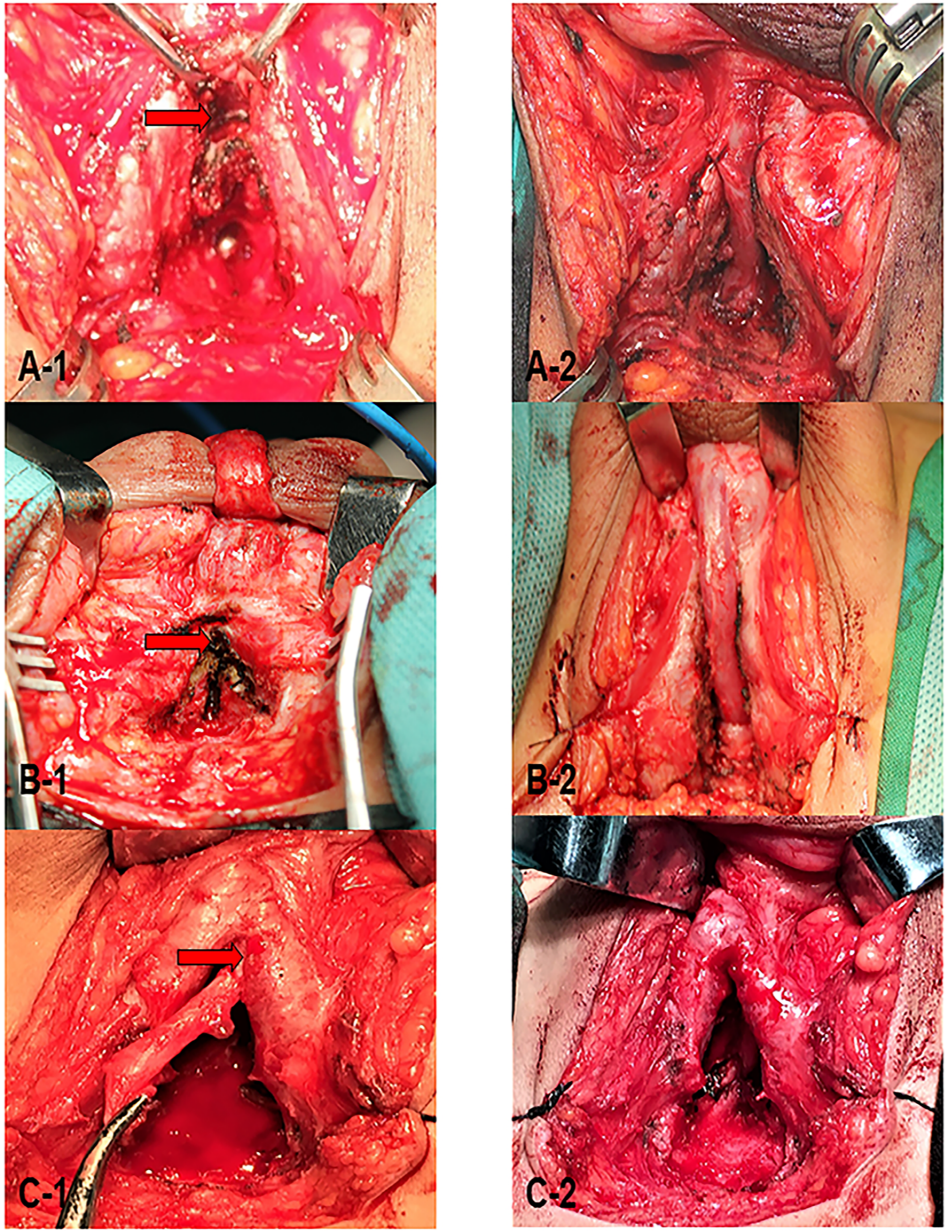
During the operation, on the basis of freeing the anterior urethra, perform (**A1-2**): penile septum incision (pointed by arrow); (**B1-2**): resection of the lower edge of the pubic symphysis (pointed by arrow); (**C1-2**): urethral transposition to foot of the cavernous body of the side penis (pointed by the arrow) to achieve tension-free anastomosis of the distal and proximal urethra.

All patients underwent preoperative urethral retrograde and voiding cystourethrography and some underwent flexible cystoscopy, revealing posterior urethral strictures or atresia. The urethra of the bulbar membrane was injured in 16 patients, the urethra of the prostatic membrane was injured in 10, and bladder neck atresia was evident in 2(Figure 1). The mean stenosis length was 4.2 cm. Eight patients had floating bladders and four urethrorectal fistulae; twelve had failed urethral surgery at other hospitals. All patients underwent end-to-end urethral anastomosis *via* a transperineal, inferior pubic approach. Patients with long urethral defects were treated using a variety of surgical techniques to free the distal urethra. To achieve tension-free anastomosis of the distal and proximal urethra, 8 patients underwent splitting of the corpus cavernous septum(Figures 2A1-2), 18 partial resection of the lower edge of the symphysis pubis (Figures 2B1-2); in 2, the urethra was rerouted under a corpus cavernosum(Figures 2C1-2). The mean postoperative follow-up duration was 36.8 months. Of the 28 patients, 24 (85.7%) evidenced unobstructed urination after a single operation. The mean maximum urinary flow rate was 17.8 ml/s. Two patients required second urethral end-to-end anastomoses and subsequently evidenced normal postoperative urination. Two patients still require bladder fistulae and are awaiting further surgery at the time of writing (Table 1). Two evidenced mild urinary incontinence and remain under follow-up. Of the six patients who have reached puberty, two have self-reported ED (Table 2).

**Table 1:** Demographics and clinical data of the 28 patients with complicated posterior urethral stricture.

Cases	28
Age (years old)	6.3 (2–14)
Length of stenosis (cm)	4.2 (3–5.5)
Location of stenosis	
Bulbar membrane	16
Prostatic membrane	10
Bladder neck	2
Operation history	
Internal urethrotomy	3
Reconstruction of ruptured urethra	4
Urethral anastomosis	5
Complicated rectal fistula	4
Operation methods	
Distal urethra free	28
Penile septum split	28
Resection of the lower edge of the pubic symphysis	18
Urethral rerouted under one corpus cavernosum	2
Operation time (min)	114.3
Bleeding (mL)	110.7
Follow-up (month)	36.8 (4–72)
Curative effect	
Succeed	85.7% (24)
Maximum flow rate (mL/s)	17.8 (15–22)
Second operation	2
Waiting for another operation	2
Complications	
Mild urinary incontinence	4
Erectile Dysfunction	3

**Table 2:** The outcome of follow up for the complications.

Patient	Stricture location and length	Age at injury(y) and interview(y)	Operation type	Short-term complications	Long-term complications
1	Bulbar membrane/3 cm	14/20	Anastomosis/ Resection the pubic symphysis	none	none
2	Bulbar membrane/4 cm	14/18	Anastomosis/ Urethral rerouted Under one corpus cavernosum	none	Mild ED
3	Bulbar membrane/3.5 cm	13/19	Anastomosis/ Resection the pubic symphysis	none	none
4	Prostatic membrane/3 cm	12/18	Anastomosis/ Penile septum split	Stricture recurrence	none
5	Prostatic membrane/4.5 cm	14/19	Anastomosis/ Resection the pubic symphysis	none	Mild ED
6	Prostatic membrane/4 cm	13/19	Anastomosis/ Penile septum split	none	none
7	Bladder neck /3 cm	3/7	Anastomosis/ Resection the pubic symphysis	none	Mild urinary incontinence
8	Prostatic membrane/3.5 cm	5/8	Anastomosis/ Resection the pubic symphysis	none	Mild urinary incontinence

## Discussion

Posterior urethral distraction defects in children are complex and difficult to manage. Many principles and techniques used in the repair of these urethral strictures in children are similar to those used in adults but in fact these verity, complexity, and nature of tissue are different in children. A consensus on the optimal treatment of these traumatic distraction defects in children is yet to be reached (5). Moreover, there was few studies about the long-term follow-up for the postoperative complications.

In children with traumatic posterior urethral strictures, both retrograde and voiding cystourethrography are routinely performed before surgery to determine the location and length of the posterior urethral stricture or stenosis. However, such cystourethrography may overestimate the urethral stenosis length, particularly in children who may not be fully cooperative; in whom the distal urethra is not fully developed. It is difficult to accurately determine the urethral stenosis length (6). We believe that a flexible cystoscopy examination is important during the preoperative evaluation of a urethral stricture. Especially in pediatric patients with incomplete bladder neck openings, use of a flexible cystoscopy allows the clinician to accurately locate the proximal urethra and estimate the urethral stricture length. Pelvic magnetic resonance imaging also accurately determines the location and length of a posterior urethral stenosis or atresia (7).

The currently accepted method for the treatment of a posterior urethral stricture is urethral end-to-end anastomosis. During the operation, the stricture and surrounding scars must be completely removed and the urethral mucosa must be exposed, enabling the distal and proximal urethra to be anastomosed without tension. The key to successful surgery is full exposure of the proximal urethral stricture to enable the surgeon to perform the urethral anastomosis under direct vision (8). Three methods are currently used for urethral end-to-end anastomosis: a simple transperineal approach, a transperineal inferior pubic approach, and a combined transpubic-perineal approach. Traditional transperineal end-to-end anastomosis is generally appropriate for patients with posterior urethral strictures less than 3 cm in length and no comorbidity. The transpubic approach is appropriate for patients with posterior urethral strictures with stenoses longer than 3 cm, stenoses close to the bladder neck, a concomitant urethral fistula or bladder neck tear, and/or who have failed multiple perineal repair surgeries. Pierce was the first to perform posterior urethral repair *via* resection of the pubic symphysis (9). The reported success rates of transperineal and transpubic posterior urethral anastomoses in pediatric patients are 80% and 100% respectively, suggesting that retropubic urethroplasty is appropriate for children with posterior urethral stricture (10). However, although this method usefully exposes the entire surgical field and affords a large surgical space, it destroys pelvic stability, associated with a high incidence of postoperative pelvic tilt that may cause low back pain, gait instability, and other complications in growing children. Therefore, retropubic urethroplasty is not currently advocated as a treatment for pediatric urethral stricture (11).

Huang et al. were the first to use partial, pubic symphysis resection to perform posterior urethral repair in China (12). This approach fully exposes the prostate and membranous urethrae and enables full removal the scar tissue around the urethra. Thus, the tension of the anterior and posterior urethral anastomoses are greatly reduced and the integrity of the pelvic ring preserved, preventing postoperative complications such as an abnormal gait and chronic low back pain. We have learned from this surgical method. During surgery, we perform wedge-shaped excision of the lower edge of the pubic bone to fully expose the proximal urethra and create space for the operation. Furthermore, incision of the penile cavernous septum and the lower edge of the pubis enables the distal urethra to pass through the incised septum, which shortens the distance between the distal and proximal ends of the urethra by about 3 cm to ensure a tension-free anastomosis; this also avoids the need for excessive freeing of the distal urethra which shortens the penis. Given the developmental characteristics of children, there are several technical points to consider during surgery. First, as children lack mature bones, the lower edge of the pubic bone is rather flexible. Although this edge must be excised when treating adults, part of the lower border of the pubic bone can be directly removed with an electric knife in children, causing less bleeding and obviating any need to replant removed bone fragments in the defect. Second, the blood vessels linking the urethra and the corpus cavernosum are small in prepubertal children, associated with a poor blood supply to the distal end of the urethra. When freeing the distal end of the urethra, the surgeon must be careful to minimally damage the communicating blood vessels. Therefore, for children with complicated posterior urethral strictures, we prefer to approach *via* the lower edge of the perineum-pubic symphysis.

**Figure 3:**
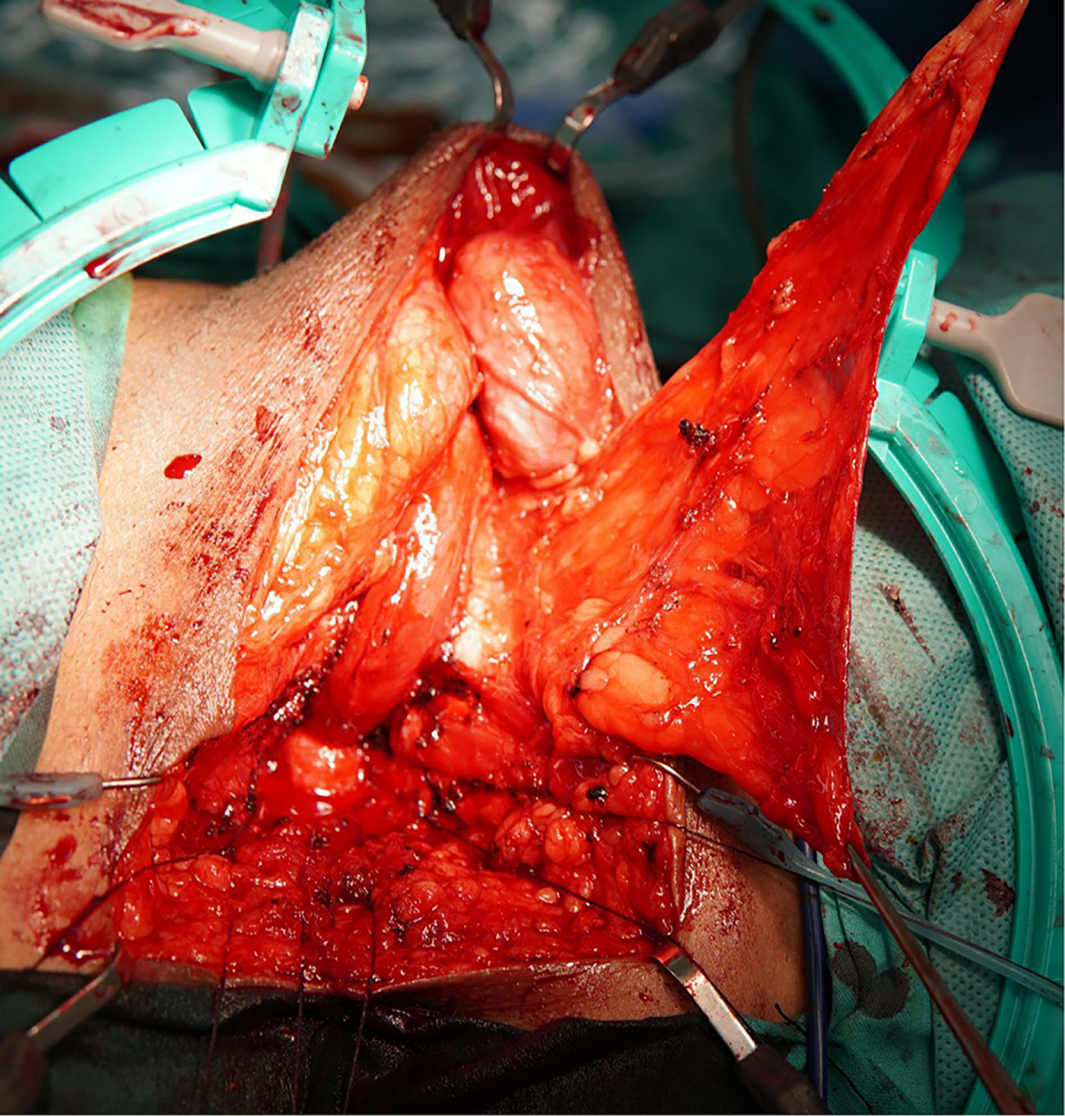
The harvest of the pedicel scrotal membrane flap.

Children with posterior urethral stenosis have often undergone multiple urethral repairs and exhibit severe scarring, creating lengthy posterior urethral defects. Given the unique nature of tissue in the posterior urethral region, urethral substitutes are usually not employed to treat such stenoses. The gold standard is resection of the stenotic urethra accompanied by tension-free anastomotic urethroplasty (13). However, in patients with complicated, long, posterior urethral strictures, tension-free anastomosis of the distal and proximal ends of the urethra cannot be achieved *via* conventional methods (such as penile septal incision and partial resection of the lower edge of the pubic symphysis after removal of the narrowed urethra). In such a situation, we surgically remove the narrow urethra and surrounding scar tissue and then reroute the urethra under one penile corpus cavernosum, followed by end-to-end anastomosis with the proximal urethra (14). This shortens the length required for tension-free end-to-end anastomosis. When the anterior urethra bypasses the corpus cavernosum on one side and then anastomoses with the urethra of the prostate, this does not pull on (bend) the corpus cavernosum, which is especially important in children, and does not affect corpus cavernosum development (15). However, care is required during operation. The anterior urethra must be adequately separated to prevent tension in the anastomosis, and the gap between the feet of the penile cavernous body on one side must be able to accommodate (loose) passage of the anterior urethra. Further, any scar tissue around the end-urethral anastomosis must be maximally removed to fully expose the urethral mucosa. After end-to-end urethral anastomosis, if a large gap is evident in the lower edge of the pubic bone, the pedicel scrotal membrane flap can be placed in the gap to eliminate dead space and increase the local blood supply (Figure 3). If both a urethral stricture and a urethral fistula are present, the flap is placed between the urethral and rectal anastomoses to form a barrier between the urethra and rectum. This promotes fistular healing and prevents infection (16). We placed the pedicel scrotal membrane flap in all four patients with urethrorectal fistula.

Long-term follow-up of posterior urethral stricture repair focuses on urinary incontinence and ED. Incontinence reflects poor integrity of the bladder neck and the severity of pelvic fracture, thus not directly related to the operation of urethral stricture (17). With the exception of cases with severe damage to the bladder neck, urinary incontinence after posterior urethral stricture repair generally resolves at least partially over time (18). The risk of postoperative incontinence can be predicted by preoperative urethrography and the integrity of the bladder neck.

ED is a major complication of vascular and nerve bundle injuries caused by traumatic pelvic fractures. The incidence of new ED in adults after pelvic fractures is 34%. Urethral surgery increases this by 3% (19). In children, ED obviously cannot be evaluated prior to puberty. Efforts have been made to predict ED. Koraitim et al. identified four predictive factors: The type of pelvic fracture, any pubic deformity, prostate displacement, and the length of the bulbar urethral defect (20). Of these, transverse prostate displacement and the length of the bulbar urethral defect were most important. When both are present, the ED nears 100%. When the urethral defect is longer than 2.5 cm, the ED incidence is significantly increased. Lateral prostate movement is generally only a few millimeters, but increases ED four-fold. Eight of our children are now adults. We telephoned them; six reported no obvious ED and two some ED. The urethral defect lengths of the latter were longer than 3.5 cm, consistent with this theory.

## Conclusion

End-to-end urethral anastomosis *via* a transperineal inferior pubic approach is an ideal treatment for complicated posterior urethral strictures in boys. This fully exposes the posterior pubic space without disrupting pelvic continuity. Based on the extent of dissociation of the distal urethra, penile septal incision, partial resection of the lower edge of the pubic symphysis, and rerouting of the urethra under a penile corpus cavernosum, can successfully handle a long posterior urethral defect. However, it is important to ensure that the free distal end of the urethra is not excessive. This not only protects the penis but also reduces urethral necrosis attributable to an insufficient blood supply. The complications include incontinence and ED; long-term follow-up is required. Especially when the patients are fully adult, we will use the questionnarie to complete the follow-up to obtain satisfied follow-up results.

## Data Availability

The original contributions presented in the study are included in the article/Supplementary Material, further inquiries can be directed to the corresponding author/s.
